# Advances in Wearable Sensors: Signalling the Provenance of Garments Using Radio Frequency Watermarks

**DOI:** 10.3390/s20226661

**Published:** 2020-11-20

**Authors:** Javad Foroughi, Farzad Safaei, Raad Raad, Teodor Mitew

**Affiliations:** 1 School of Electrical, Computer and Telecommunications Engineering, Faculty of Engineering and Information Sciences, University of Wollongong, Wollongong, NSW 2522, Australia; raad@uow.edu.au; 2 Westgerman Heart and Vascular Center, University of Duisburg-Essen, 45122 Essen, Germany; 3 School of the Arts, English and Media, University of Wollongong, Wollongong, NSW 2522, Australia; tmitew@uow.edu.au

**Keywords:** provenance, wearable sensors, garment, watermarks, radio frequency, smart textiles, traceability, chipless RFID

## Abstract

There is a significant nascent market for ethically produced products with enormous commercial potential around the world. A reliable method to signal the provenance of products is therefore critical for industry, given that competition based on price is not a viable strategy. The ability to trace and signal ethical treatment of animals is also of significant value to textiles manufactures. The efficacy of such a method can be measured with respect to the cost of implementation, scalability, and the difficulty of counterfeiting. The key to traceability is to win the trust of the consumer about the veracity of this information. Wearable sensors make it possible to monitor and improve the management of traceability and/or provenance. In this paper, we introduce a method for signalling the provenance of garments using radio frequency watermarks. The proposed model consists of two levels of authentication that are easy to use by legitimate vendors, but extremely difficult to imitate or hack, because the watermark is built-in and based on the radiation signature of electroactive materials.

## 1. Introduction

The notion of provenance stands for the process of establishing and authenticating a record of origin, as well as the logistics of production, distribution, and usage of a given product. Moreover, the ability to map and access logistical information about a product gives us a level of guarantee for ethical and certified production processes [1].

**Figure 1:**
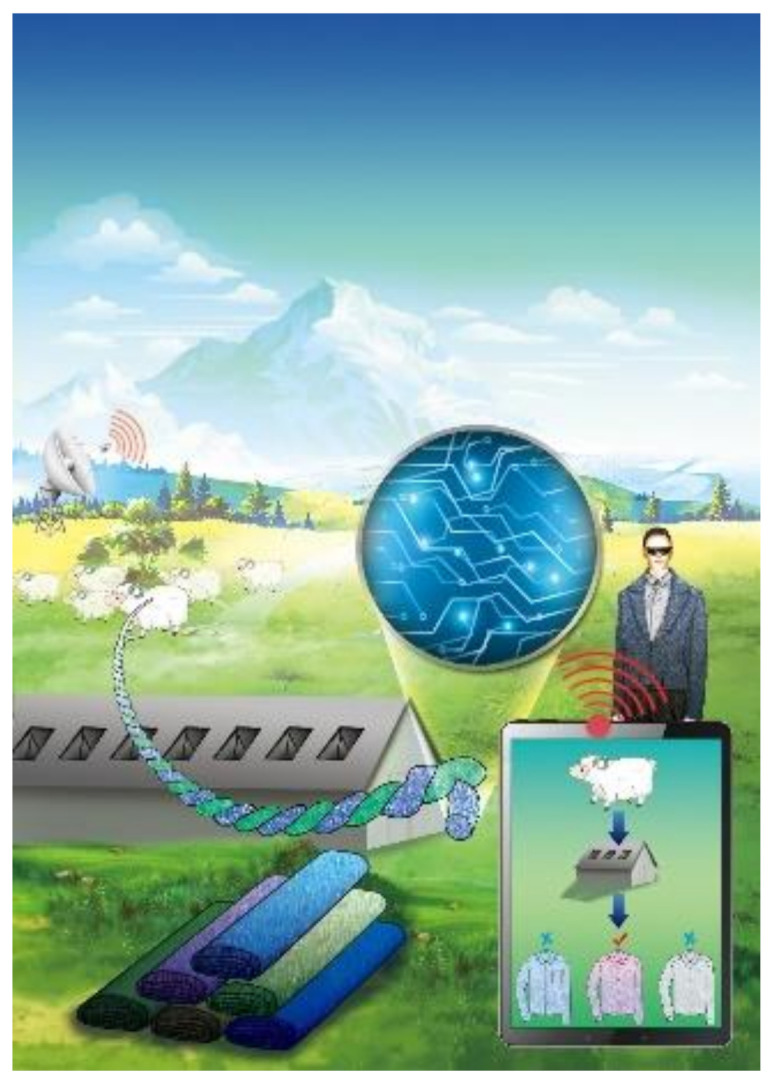
Schematic of proposed wearable sensors for authenticating provenance using radio frequency watermarks.

There is growing evidence in many industries, including garments, that certain market segments attach value to this information. In Australia, for example, signalling the authenticity of Australian beef, honey, or wheat has attracted significant effort by the industry. The Responsible Wool Standard (RWS) introduced by the World Textile Exchange has a similar aim, requiring that each stage of wool production is certified, allowing the producer to establish and maintain a globally distinctive brand position as a sustainable and socially responsible producer. This specific context makes incorporation of provenance within the fabric particularly important, as it allows producers to differentiate themselves from (cheap) imitations and tap into a socially and environmentally conscious market segment that is likely to show significant loyalty to the brand. The process can conceptually be visualized as consisting of two distinct phases: *establishing* provenance and *authenticating* it [2,3,4,5]. For example, in the context of the wool industry, the establishing phase allows a wool producer to map and follow the entire logistical chain from animal to distributor, while the authentication phase incorporates a method to *signal* its provenance and for distributors and customers to be able to *verify* this signal (Figure 1).

## 2. Signalling Provenance

The establishment phase of provenance often involves a lengthy process of verification for each stage in the production and logistic chain. In the case of wool garments, for example, this involves incorporation and verification of all available data about the following:

1- The source animal: date and place of birth, conditions of life.
2- The producer: location, labour practices, ethical treatment of animals, supply chain.
3- The processor and distributor: location, labour practices, quality of process, supply chain.
4- The consumer: location, wearing patterns, etc.

Traditionally, this phase is labour intensive, involves lots of paperwork and site visits, and is performed by certification authorities that are publicly or privately owned. There has been a surge of innovation in this space, both by the incumbents and new start-ups to improve the efficacy of this phase with respect to the cost of implementation and scalability. Typically, this is achieved by adopting new technology for labelling, tagging, and tracking, plus a distributed and trusted ledger.

The certification authority must be trusted by the customers. The emergence of trust-less distributed ledgers, such as blockchain, may represent a scenario in which the involvement of a trusted organization is no longer mandatory. It is, however, debatable whether it is possible to have a fully trust-less system. For example, even if the blockchain method is shown to be fool proof, it is necessary, as a minimum, to trust its software implementation [6]. However, when it comes to provenance, the main problem of trust is not so much with the certification authority per se but the way provenance is *signalled*. Using garments as an example, the state-of-the-art methods for signalling provenance are labelling, printing, external devices, and by weaving onto the fabric [7,8]. In labelling, a small piece of a paper, fabric, plastic, or similar is attached to the garment. In printing, an image or a logo is directly printed onto the garment with the associated information. In the case of devices, radio-frequency identification (RFID) tags or electronic devices are attached. Finally, in the case of weaving, a logo or label is made of interlacing different threads, yarns, strips, or fibrous material with each other.

Trusting these signals may be problematic for a consumer given the ease with which counterfeiting can be achieved. An Australian consumer, for example, may not have great difficulty in trusting the Australian Certified Organic (ACO) standard and the underlying organization. Nevertheless, there are at least two difficulties when the ACO logo is printed on the packaging of a product: First, is the logo genuine? Second, does the organization have the necessary resources to monitor and eliminate all inappropriate use and imitations of such logo?

The granularity of information provided by a genuine logo is also coarse and its signal blunt, meaning that it is not specific to the product or artefact being purchased. For example, if the food product is imported, the customer may have greater difficulty believing that an Australian standard can be fully imposed on a producer residing in another country.

A reasonably well-trusted traditional technique for signalling provenance is a watermark. Given that the pattern of a watermark on paper has to be produced during the paper fabrication phase, counterfeiting is generally more difficult. Digital watermarking has taken this to the realm of digital signals by embedding hidden digital information in audio, image, or video files to identify ownership.

To adapt this concept for signalling provenance, a *radio frequency watermark* can be embedded into the fabric. The watermark in this case will be the radiation signature of a (hidden) pattern of antennas incorporated into the fabric or the packaging of other goods. The aim is to develop a system that is easy to use by legitimate vendors, but extremely difficult to imitate or hack. The RF watermark can be *specific* so as to achieve sufficient granularity in signalling provenance, and it can be *flexible* to allow adoption of a variety of business models and innovative marketing strategies.

## 3. Radio Frequency Watermark

Near-field communication through RFID is a well-known technology, consisting of a simple chip with a coil acting as a near-field antenna. There is no battery in the device and all energy is obtained from the coupling of the near-field provided by the reader. The reader could be a standalone device or incorporated into a mobile phone.

The proposed RF watermark is essentially a chipless RFID that is woven into the fabric or integrated into the packaging material. The single-sided chipless RFID tag that has been developed can be easily printed on paper or plastic packets based on a low cost printable slot-loaded dual-polarized tag with four near- and far-field reading techniques [9,10,11]. In another study, the authors study the effect of deformation of a tag on clothing (bending) [12], which may lead to false detection, and it is concluded to place the tag on a flat region of the body [13]. Furthermore, a tag was made of three dual rhombic loop scatters encoded with 3 bits capacity [14]. To the best of our knowledge, the existing works are endeavouring to print the watermark onto the clothing using standard material and there is no integration into the yarn itself.

In our proposed RF watermark, the information is encoded as a combination of the arrangement pattern of textile antennas within the fabric and a specified radiation signature resulting from this arrangement and electromagnetic properties of the yarns. This allows the user to authenticate the origin of the fabric and its provenance because the proof is built into the fabric itself and thwarts counterfeiting.

The amount of information that can be communicated to the reader using an RF watermark is quite small (a few bits). Furthermore, the pattern is built into the fabric, so the information cannot be rewritten or altered post-production. On the other hand, imitation or counterfeiting is extremely difficult or impossible. Therefore, this level of authentication is trustworthy but limited in functionality. For a complete commercial application, a chip-based RFID can be incorporated in a suitable manner, either woven or attached, to the *garment*. The garment authenticity is in tandem with fabric authenticity but may contain different information (for example, the garment manufacturer may not be the same as the fabric producer). However, without the authentic fabric, the garment authentication process is incomplete and cannot be trusted. In other words, this second level of authentication builds on the security of the first method but provides more functionality. The combination of the two, therefore, provides the necessary solution for verifiable signalling of provenance.

## 4. Technical Challenges and Current Research

The key technical challenges for the development of an RF watermark can be summarized as follows:

1- The development of wearable or textile antennas with suitable gain, directivity, and bandwidth that do not compromise the wearer’s comfort. The textile antenna must be small, thin, lightweight, robust, inexpensive, and easily incorporated in cloths.
2- For fabric-based antennas, the system has to deal with the effects of deformation and strain on performance. During normal usage, the fibres endure not only bending and twisting, but are also exposed to mechanical strain and elongation. All these factors will have a significant effect on the radiation pattern of the antennas.

We now briefly discuss the state of the art with respect to these two topics.

### 4.1. Textile Antennas

Wired antennas are the simplest and oldest type of antennas. In smart fabrics, monopole and dipole antennas are usually preferred for wireless communication because of their omnidirectional radiation patterns, which makes the antenna independent of its rotation. However, the disadvantage of these antennas is that they are oriented perpendicular to the body surface, hence making them uncomfortable and obtrusive. A microstrip patch antenna comprises of a radiation patch at one side of a dielectric substrate and a ground plane on its other side, and it may be suitable candidate for textile antennas. Its advantage is that it radiates perpendicular to the planar structure and the ground plane helps to mitigate the effect of human body tissues. Moreover, the low volume, planar configuration, lower cost, and low weight make it a suitable contender for integration into the fabric. Therefore, the crucial elements of designing wearable and textile antennas are planar structures, flexible conductive materials for the patch, the ground plane, and the flexible dielectric materials as a substrate. Specifically, the conductivity of the ground plane and patch plays an important role to determine the efficiency of the antenna and it should be as high as possible. Similarly, the permittivity and thickness of the dielectric substrate determine the bandwidth and gain of the antennas.

Currently, polymer-based antennas have been developed for lightweight and flexible applications. The methods to develop flexible antennas include printing and laminating of graphene ink on papers, using graphene-based antennas on polymeric substrates, and etching copper film geometries on polymeric substrates. Although these devices are bendable and flexible, the substrates have some limitation to be utilized for large mechanical strain and elongation applications. In addition, polymeric substrates develop other issues for the garment’s heat and moisture management due to the creation of a sealing and non-breathable layer on the skin. To overcome these issues, the antennas should be directly incorporated in or onto garments. Consequently, textiles antennas enable the flexibility and porosity of fabrics that would provide desirable requirements for the ideal garments; such garments would be soft, deformable, breathable, and protective for the skin [11,15]. In addition, the antennas can be concealed, which improves the user experience. The challenge is to develop electrically conductive fibres that are compatible with mechanised textile processing such as weaving, knitting, and braiding. Fine metallic wires can be used, but the textiles have an undesirable rigidity that is uncomfortable in clothing. Metal-coated polymer yarns, such as silver-coated nylon, are commercially available; however, when very thin yarn is used, it becomes a challenge to emit an electromagnetic wave. In addition, the coating is subject to deterioration.

**Figure 2:**
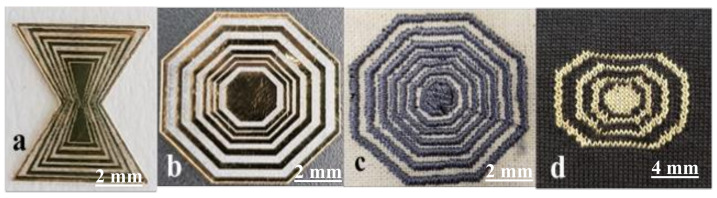
Prototype chipless radio frequency identification (RFID) tags recently developed by our team, (**a**,**b**) fabricated bow-tie and octagonal chipless RFID tags based on Gold mylar using a laser etching technique, (**c**) embroidered RFID tag using conductive yarn (silver-coated nylon), and (**d**) knitted RFID tag using a conductive yarn and knitting technique [16].

**Figure 3:**
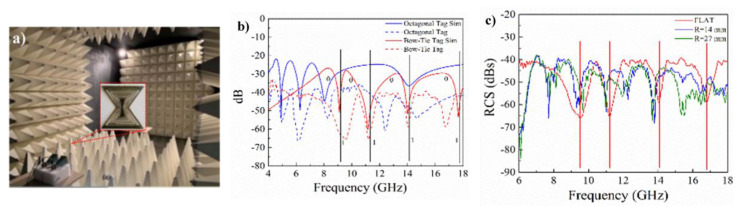
Our developed chipless RFID tags [16], (**a**) experimental set-up in chamber, (**b**) simulated and measured RCS responses of octagonal and bow-tie chipless RFID tags, and (**c**) Bending of chipless bow-tie RFID tag with polyethylene terephthalate (PET) substrate at R = 27 mm and R = 14 mm.

We have demonstrated a novel RF watermark based on conductive silver-coated nylon yarn for the first time using embroidery and knitting techniques. Figure 2 and Figure 3 show some of the initial developments and test results by our team in this regard [16]. In Figure 2, an example octagonal and bow-tie antenna pattern is created, both in gold and without defects for benchmarking, and it is also embroidered and knitted on and into the fabric to examine the effects of imperfections. Figure 3a shows the test set up in the anechoic chamber to carry out the experiments. The comparison between the measurement radiation signature against simulation for two antenna patterns of bow-tie and octagonal shapes are shown in Figure 3b. There is reasonable agreement between simulation and experiments and, in particular, the ‘dips’ are well aligned. The location of these dips can be tuned based on the antenna design and these can be considered as encoding the information [17].

### 4.2. Fabric Deformation and Strain

To counter the effects of fabric deformation and strain, two approaches are being pursued. Firstly, by using zigzag stiches during the embroidery process [18], the strain will be distributed evenly during elongation and stretching. In addition, by introducing a sine-wave modulation in the conventional spiral coil, the geometry deforms evenly in all directions with the least strain [19]. We have also demonstrated the effect of different bending on the bow-tie chipless RFID tag in Figure 3c.

**Figure 4:**
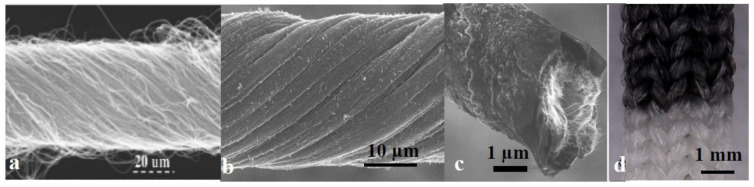
(**a**) Twisted carbon nanotube yarn, (**b**) highly conductive hybrid carbon nanotube/graphene yarn, (**c**) hybrid conducting polymer carbon nanotube, and (**d**) developed carbon nanotube knitted fabric as wearable sensors and actuator (Reproduced from Ref. 22 with permission from The Royal Society of Chemistry) [19,20,21,23].

Electroactive fibres, including conducting polymers, carbon nanotubes (CNT), and graphene yarns would be promising candidates for textiles antennas because of their similarity in mechanical properties to conventional fibres and yarns, as shown in Figure 4. These materials, either in pristine form or in combination with other polymers, metals, or nanoparticles, exhibit superior mechanical and electronic properties [20,21,22,23]. We have recently reported a means for fabricating electroactive CNT-spandex stretch fabrics by a minor modification of a commercial circular knitting machine [24]. The textile created from the yarn is very stretchy and capable of conducting electricity. The knitted CNT/spandex fabric electrical conductivities were tailored in the range of 870 to 7092 S/m. The knitted CNT fabric (Figure 4d) allowed us to develop the first generation of smart clothing that simultaneously monitors the wearer’s movements, senses strain, and adjusts the garment to support or correct the movement.

**Figure 5:**
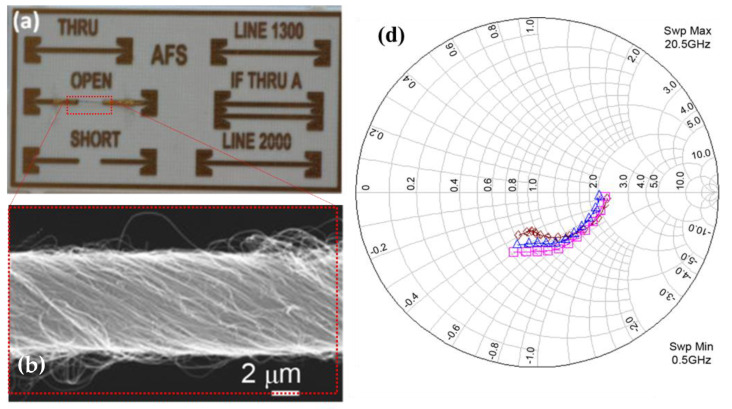

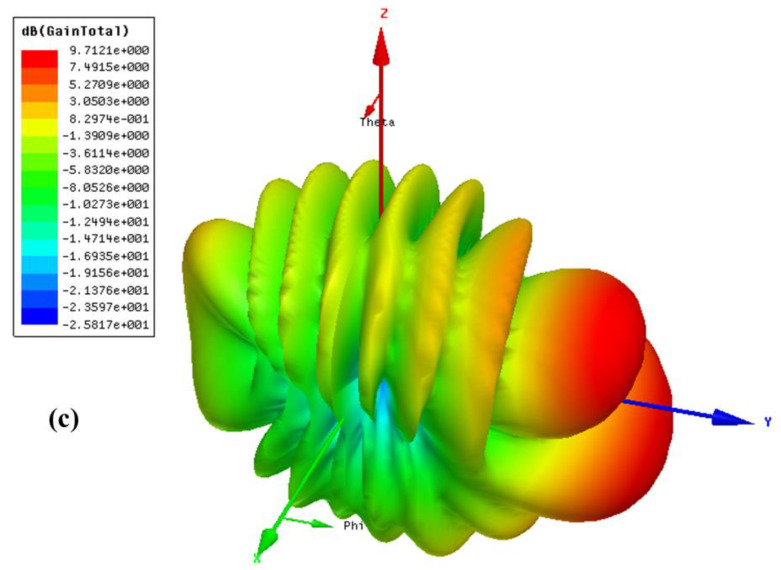
Microwave characterization of carbon nanotube yarns for ultra wideband( UWB) medical wireless body area networks, (**a**) a photo of the test structure with calibration structures, (**b**) SEM micrographs of carbon nanotube (CNT) yarn sample attached to golden pads (scale bar = 2 μm), (**c**) patch antenna radiation pattern, and (**d**) measured S21 for the three 100-μm CNT yarns on 3500-μm substrates showing moderate absorption in transmission and resistance at dc [24].

In addition, the high frequency properties of multiwalled carbon nanotube yarns in the GHz range have been studied (Figure 5). We have shown that multiwalled carbon nanotube yarns can be utilized as textiles antennas for body area networking. We demonstrated multiwalled carbon nanotube fibres as potential data transmission lines. Scattering parameters of multiwalled carbon nanotube yarns were measured from 50 to 20 GHz and shown on the Smith chart in Figure 5d [21,22,24]. The obtained results shown that frequency-independent resistive behaviour of the multiwalled carbon nanotubes is a very promising indicator due to its added values of durability and thermal conductivity which are suitable for a range of applications such as body area networks [25].

## 5. Future Outlook

High-performance flexible textiles antennas with high electrical conductivity that are thin, lightweight, robust, inexpensive, and easily incorporable to clothes will be of benefit for future wearable technologies. The development of textiles antennas, however, is still a challenge. The processes of textile antennas for wearable sensors could be associated with several challenges, such as the flexibility and porosity of fabrics. In addition, electrical shorting between antennas at their mechanical intersection is another key limitation generated due to damage of electrical connections during washing and other everyday activities. Consequently, it is necessary to protect electronic textiles using hydrophobicity and electrical encapsulation through surface modification of the textile antenna. For example, to eliminate these issues, the textile antenna could be coated with silicone rubber to minimize the limitations described above. Recently, a method of encapsulation for washable, reliable, and wearable electronics has been demonstrated using two types of silicone where the devices were able to perform after washing. To this end, controlling a number of factors, such as durability, flexibility, washability, and electrical conductivity during manufacturing, wearable textiles antennas should be considered in order to produce high-performance wearable sensors [26,27].

## 6. Conclusions

The manufacturing, counterfeiting, and selling of trademarked and branded merchandise presents a substantial threat to producers, as it leads to revenue loss and the deterioration of customer perception of value and uniqueness of a brand. There is a significant nascent market for authentic, environmentally friendly, and ethically produced products. The RF watermark represents a reliable method to signal provenance and is therefore critical for such businesses, given that competition based on price would not be a viable strategy. Importantly, the solution is applicable to both synthetic and natural fibre garments, as well as a wide range of packaging. It will provide a system that is extremely difficult to imitate or hack, because the fabric provenance is built-in using radiation signatures of electroactive yarns.
